# CX3CL1 induces cell migration and invasion through ICAM‐1 expression in oral squamous cell carcinoma cells

**DOI:** 10.1111/jcmm.17750

**Published:** 2023-04-21

**Authors:** Chia‐Yu Wu, Pei‐Wen Peng, Ting‐Yi Renn, Chia‐Jung Lee, Tsung‐Ming Chang, Augusta I‐Chin Wei, Ju‐Fang Liu

**Affiliations:** ^1^ School of Dental Technology, College of Oral Medicine Taipei Medical University Taipei City Taiwan; ^2^ Division of Oral and Maxillofacial Surgery, Department of Dentistry Taipei Medical University Hospital Taipei City Taiwan; ^3^ Department of Oral and Maxillofacial Pathobiology, Graduate School of Biomedical and Health Sciences Hiroshima University Hiroshima Japan; ^4^ Department of Otolaryngology Head and Neck Surgery Shin‐Kong Wu‐Ho‐Su Memorial Hospital Taipei City Taiwan; ^5^ School of Medicine Fu‐Jen Catholic University Taipei City Taiwan; ^6^ Institute of Physiology, School of Medicine National Yang Ming Chiao Tung University Taipei City Taiwan; ^7^ Translational Medicine Center Shin‐Kong Wu Ho‐Su Memorial Hospital Taipei City Taiwan; ^8^ School of Oral Hygiene, College of Oral Medicine Taipei Medical University Taipei City Taiwan; ^9^ Department of Medical Research China Medical University Hospital, China Medical University Taichung City Taiwan

**Keywords:** CX3CL1, human oral squamous cell carcinoma (OSCC), ICAM‐1, PLCβ/PKCα/c‐Src pathway tumour motility

## Abstract

Human oral squamous cell carcinoma (OSCC) has been associated with a relatively low survival rate over the years and is characterized by a poor prognosis. C‐X3‐C motif chemokine ligand 1 (CX3CL1) has been involved in advanced migratory cells. Overexpressed CX3CL1 promotes several cellular responses related to cancer metastasis, including cell movement, migration and invasion in tumour cells. However, CX3CL1 controls the migration ability, and its molecular mechanism in OSCC remains unknown. The present study confirmed that CX3CL1 increased cell movement, migration and invasion. The CX3CL1‐induced cell motility is upregulated through intercellular adhesion molecule‐1 (ICAM‐1) expression in OSCC cells. These effects were significantly suppressed when OSCC cells were pre‐treated with CX3CR1 monoclonal antibody (mAb) and small‐interfering RNA (siRNA). The CX3CL1‐CX3CR1 axis activates promoted PLCβ/PKCα/c‐Src phosphorylation. Furthermore, CX3CL1 enhanced activator protein‐1 (AP‐1) activity. The CX3CR1 mAb and PLCβ, PKCα, c‐Src inhibitors reduced CX3CL1‐induced c‐Jun phosphorylation, c‐Jun translocation into the nucleus and c‐Jun binding to the ICAM‐1 promoter. The present results reveal that CX3CL1 induces the migration of OSCC cells by promoting ICAM‐1 expression through the CX3CR1 and the PLCβ/PKCα/c‐Src signal pathway, suggesting that CX3CL1‐CX3CR1‐mediated signalling is correlated with tumour motility and appealed to be a precursor for prognosis in human OSCC.

## 1 INTRODUCTION

Oral cancer is the most common type of head and neck squamous cell carcinoma (HNSCC), the most prevalent malignancy worldwide.^1^ Human oral squamous cell carcinoma (OSCC) accounts for most oral cancers and takes various clinical forms with high mortality and morbidity rates, resulting from the cancer metastasis and locoregional recurrence.^2^, ^3^ Its survival rate is strongly correlated with the TNM (tumour size, nodal metastasis and distant metastasis) stage of the primary tumour.^4^ Even thoughdiagnostic techniques and treatment modalities are advanced for OSCC, its 5‐year relative survival rate is less than 50% for advanced‐stage oral SCC because of large lesions and clinically detectable metastases to cervical lymph nodes at the time of diagnosis.^5^ Thus, understanding and recognizing the molecular mechanism at the early stage is critical for prognosis of patients with OSCC.

C‐X3‐C is a chemokine family member, primarily affecting leukocyte migration and tumour growth and development.^6^ C‐X3‐C motif ligand 1 (CX3CL1) is a multifunctional inflammatory chemokine that plays a central role in the pathogenesis of inflammation‐driven malignancies.^7^ CX3CL1 mediates immune cell adhesion in membrane‐anchored form and acts as a chemotactic cytokine in the soluble form.^8^ Studies also demonstrated that CX3CL1 and its unique chemokine receptor CX3CR1 played an essential role in cancer development. CX3CL1‐CX3CR1 axis regulates numerous cellular functions relevant to cancer survival mechanisms, including proliferation, migration, invasion and apoptosis resistance.^9^, ^10^, ^11^ However, contradictory data exist concerning the role in tumour progression in the specific cancer type for CX3CL1‐CX3CR1.^7^, ^12^, ^13^, ^14^ The CX3CL1‐CX3CR1 axis was reported to predict better prognosis and fewer recurrences in hepatocellular carcinoma.^8^ In contrast, activation of the CX3CL1‐CX3CR1 axis promotes various neoplasia responses, including tumorigenesis and progression in ovarian carcinoma and gastric, pancreatic and lung cancer.^15^, ^16^, ^17^, ^18^ Nevertheless, the investigation of the stimulatory effects of CX3CL1 on the tumour cell motility and progression in OSCC cells at signalling mechanism and gene expression levels was limited.

Metastasis is associated with multiple steps in which tumour cells depart from the primary tumour site and migrate to other secondary locations through the bloodstream, lymphatic system or direct extension.^19^ CX3CL1 promoted cell migration and lung metastasis in human osteosarcoma by upregulating intercellular adhesion molecule‐1 (ICAM‐1) expression.^20^ The CX3CL1/ICAM‐1 signalling network facilitated cell adhesion and transendothelial migration and initiated spinal metastasis in non‐small‐cell lung cancer.^21^ ICAM‐1, a transmembrane glycoprotein in the immunoglobulin superfamily, is constitutively expressed at a low basal level in vascular endothelial and epithelial cells, but its expression is upregulated for the stabilization of cell–cell interactions and endothelial transmigration during inflammation.^22^, ^23^, ^24^ The expression of ICAM‐1 reveals the correlation between the immune system and tumour growth, even though the pro‐ or anti‐tumour immune roles of ICAM‐1 are controversial.^25^, ^26^, ^27^ ICAM‐1 exhibits upregulated expression in malignant conditions, potentially participating in tumorigenesis and promoting the metastatic ability of melanoma, breast, gastric, pancreatic and lung cancers.^28^, ^29^, ^30^ In contrast, some studies revealed that upregulation of ICAM‐1 expression reflected low growth potential and good prognosis in breast, gastric and colorectal cancers.^31^, ^32^, ^33^, ^34^

In this study, we evaluated the role of CX3CL1 in the activation of ICAM‐1 and their roles in cell movement, cell migration and invasion in OSCC. We utilized the OSCC cell lines SCC4 and SAS and found that CX3CL1 activates PLCβ, PKCα and c‐Src. Using a combination of inhibitors or siRNAs for these kinases, we found a decrease in metastasis‐associated events such as cell migration and invasion in OSCC. The data represented here contributes to the molecular characterization of the signalling events associated with CX3CL1 contributions to cell migration and invasion in OSCC.

## 2 MATERIALS AND METHODS

### 2.1 Materials

Primary antibody for VCAM‐1 (GTX110684; 1:1000) and ICAM‐1 (GTX100450; 1:1000) was acquired from GeneTex International Corporation. The p‐phospholipase Cβ (p‐PLCβ) (2481S; 1:1000), PLCβ (14247S; 1:1000), p‐protein kinase Cα (p‐PKCα) (9375S; 1:1000), cellular Src (c‐Src) (2109S; 1:1000), p‐c‐Src (5473S; 1:1000), c‐Jun (9165S; 1:1000) and p‐c‐Jun (2361S; 1:1000) were acquired from Cell Signaling Technology. PKCα (SC2081240; 1:1000) was acquired from Santa Cruz Biotechnology. β‐actin (SI‐A5441; 1:10,000) and secondary antibody for anti‐rabbit and anti‐mouse were acquired from Merck KGaA. Inhibitors for PLCβ (GF109203X; AB‐144264), PKCα (U73122; U6756), c‐Src (PP2; SI‐P0042) and c‐Jun (curcumin; C7727 and tanshinone IIA; T4952) were acquired from Sigma‐Aldrich. Specific neutralizing antibodies for CX3CL1 (AF365‐SP) and CX3CR1 (AF5825) were acquired from Novus Biologicals. Human recombinant protein CX3CL1 (300–31) was acquired from PeproTech and dissolved according to the manufacturer's manual. All other chemicals and reagents were acquired from Sigma‐Aldrich.

### 2.2 Cell lines and cell culture

Three human OSCC cell lines (SCC4, SCC25 and SAS cells) were acquired from the Bioresource Collection and Research Center (BCRC). SCC4 cells were cultured in Dulbecco's modified Eagle's medium F12 (DMEM:F12; 30‐2006) supplemented with 10% heat‐inactivated foetal bovine serum (FBS) and 400 ng/mL hydrocortisone in an atmosphere of 5% CO_2_ at 37°C. SCC25 cells were cultured in DMEM and Ham's F12 medium containing 1.2 g/L sodium bicarbonate, 2.5 mM L‐glutamine, 15 mM HEPES and 0.5 mM sodium pyruvate and supplemented with 400 ng/mL hydrocortisone and 10% FBS in an atmosphere of 5% CO_2_ at 37°C. SAS cells were cultured in 45% DMEM with 45% Ham's F12 medium and supplemented 10% FBS in an atmosphere of 5% CO_2_ at 37°C.

### 2.3 Online database analysis

The CX3CL1 expression and clinical HNSCC patient data were downloaded from The Cancer Genome Atlas (TCGA) and analysed using an interactive web resource (UALCAN, http://ualcan.path.uab.edu/index.html).^35^ Available patient survival data over 5 years were used for Kaplan–Meier survival analyses and to generate overall survival plots with high CX3CL1 expression and low/medium CX3CL1 expression. Gene expression profiles of tumour and normal samples were obtained from the Gene Expression Omnibus (GEO) dataset and analysed for levels of intercellular adhesion molecule‐1 (ICAM‐1) and vascular cell adhesion molecule‐1 (VCAM‐1) expression. Screening of the GEO datasets revealed the GSE3524, GSE13601 and GSE78060 microarray, consisting of human OSCC and adjacent noncancerous tissue samples.

### 2.4 Wound‐healing and cell movement assay

Cells were seeded in a culture insert (IBIDI; 80209) at a density of 2.5 × 10^4^ cells per well and incubated overnight at 37°C, allowing the cells to attach. Then the culture insert was removed, and non‐adherent cells were washed using phosphate‐buffered saline (PBS). The cells were incubated for 24 h with the recombinant protein CX3CL1 at three different concentrations (control, 10 and 30 ng/mL). Photographs of the wound were taken immediately (0 h) and after 24 h incubation using an inverted microscope (ECLIPSE Ti) combined with Nikon's NIS‐Elements imaging software (Version 5.02.01) and analysed using ImageJ software (1.53a). Four independent experiments were conducted.

### 2.5 Cell migration and invasion assay

Migration and invasion assay were performed using a 24‐well transwell insert chamber (8.0 μm pore size; Corning Incorporated) without and with Matrigel‐coated membrane, respectively. OSCC cells at a density of 5 × 10^4^ cells per well were seeded onto the upper chamber with 5% CO_2_ at 37°C. Approximately 500 μL of cell culture media containing 1% FBS was added to the lower chamber, which was supplemented with the recombinant protein CX3CL1 at three different concentrations (control, 10 and 30 ng/mL). After 24 h incubation, the cells migrating to the bottom chamber were fixed with 1% formaldehyde for 15 min and stained with 0.05% crystal violet for 30 min. The number of cells was captured using an inverted microscope analysed using ImageJ software. In addition, OSCC cells pre‐treated with IgG or CX3CL1 monoclonal antibody (mAb) were also used for migration and invasion assay. Four independent experiments were conducted.

### 2.6 Total RNA extraction and real‐time polymerase chain reaction (qPCR) assay

Total RNA in OSCC cells was purified using TRI reagent (9424; Sigma‐Aldrich) according to the manufacturer's instructions. The total RNA was reverse transcribed into complementary DNA (cDNA) using RT‐PCR kit (PCRBIOSYSTEM), which was analysed using KAPA SYBR FAST qPCR Master Mix and CFX Connect Real‐Time System (BIO‐RAD). The 2^−△△Ct^ method was used to calculate the relative mRNA expression levels and the level of glyceraldehyde‐3‐phosphate dehydrogenase (GAPDH) expression was used as the internal reference gene. Four independent experiments were conducted. The sequences of primers used in this assay were listed below:

ICAM‐1 (Forward: 5′‐ACCATCTACAGCTTTCCG‐3′; reverse: 5′‐TCACACTTCACTGTCACC‐3′); VCAM‐1 (Forward: 5′‐ATGCCTGGGAAGATGGTCG‐3′; reverse: 5′‐TCTGGGGTGGTCTCGATTTTA‐3′) and GAPDH (Forward: 5′‐ACAGTTGCCATGTAGACC‐3′; reverse: 5′‐TTGAGCACAGGGTACTTTA‐3′).

### 2.7 Total protein extraction and western blot assay

Total protein of OSCC cells was collected using lysis buffer containing protease inhibitor as previously described.^36^ Protein concentration was quantified using Bicinchoninic Acid Protein Assay Kit (Sigma‐Aldrich). Then, OSCC proteins were separated by using 8%–12% SDS‐PAGE, transferred to 0.45 μm polyvinylidene membranes (PVDF), blocked with 5% nonfat milk at room temperature for 1 h and incubated with primary antibodies (1:1000) at 4°C overnight. The PVDF membranes were washed with TBST three times and further incubated with secondary antibodies (1:10,000) at room temperature for 1 h. The protein bands were visualized using ECL Select™ Western Blotting Detection Reagent (Cytiva; RPN2235) and photographed using UVP ChemiDot‐It 815 Imager BioImaging System. Four independent experiments were conducted.

### 2.8 Small‐interfering RNA (siRNA) transfection assay

Human oral squamous cell carcinoma cells were transfected with specific siRNA of ICAM‐1, CX3CR1, PLCβ, PKCα, c‐Src and c‐Jun by Lipofectamine 3000 (Invitrogen) according to the manufacturer's instruction. The transfection condition was performed at 37°C in 5% CO_2_. After 24 h incubation, the culture media were replaced with 10% heat‐inactivated FBS. Then, transfected OSCC cells were treated with recombinant protein of CX3CL1 for further experiments.

### 2.9 Immunofluorescence assay

After SCC4 and SAS cells were pre‐treated with specific inhibitors for 1 h, followed by recombinant protein of CX3CL1 for 2 h. Cells were fixed with 1% formaldehyde for 30 min, permeabilized with 0.05% Triton X‐100 at room temperature for 5 min, and then blocked with buffer (Goal Bio; W‐3400) at room temperature for 1 min. Cells were incubated with primary antibody specific for c‐Jun (1:100) at 4°C overnight. Slides were washed three times with PBS and given appropriate FITC secondary antibody (1:100) at room temperature for 2 h. Then, cells were stained with DAPI (5 μg/mL) for 5 min and observed using an inverted microscope.

### 2.10 Activator protein 1 (AP‐1) luciferase assay

Human oral squamous cell carcinoma cells were seeded in a 12‐well plate for 24 h and transfected with an AP‐1 luciferase plasmid (Promega) for 24 h according to the manufacturer's instructions. Cells were treated with specific inhibitors and CX3CL1 recombinant protein for 24 h. The microplates were read for luciferase by 2030 Multilabel Reader (VICTOR™X2; PerkinElmer).

### 2.11 Chromatin immunoprecipitation (ChIP) assay

After the pre‐treatment of inhibitors for 1 h and followed by CX3CL1 for 2 h, SCC4 cell was fixed with 4% formaldehyde for 15 min to obtain protein‐DNA chromatin fragments at 200–500 bp and immunoprecipitated with c‐Jun. The final precipitated chromatin was assayed by 2% electrophoresis gel. Four independent experiments were conducted. The primer sets of AP‐1 promoter binding sites on ICAM‐1 were used to obtain the product (AP‐1 F: 5′‐AGACCTTAGCGCGGTGTAGA‐3′; R: 5′‐AGTAGCAGAGGAGCTCAGCG‐3′).

### 2.12 Statistical analysis

All results were analysed by using Sigmastat program. All the quantified results were expressed at the mean ± standard deviation (SD) and analysed with one‐way anova followed with Fisher's least significant different (LSD) post hoc test. All results were considered *p* < 0.05 as a significant difference.

## 3 RESULTS

### 3.1 Clinicopathological characteristics of CX3CL1

**FIGURE 1:**
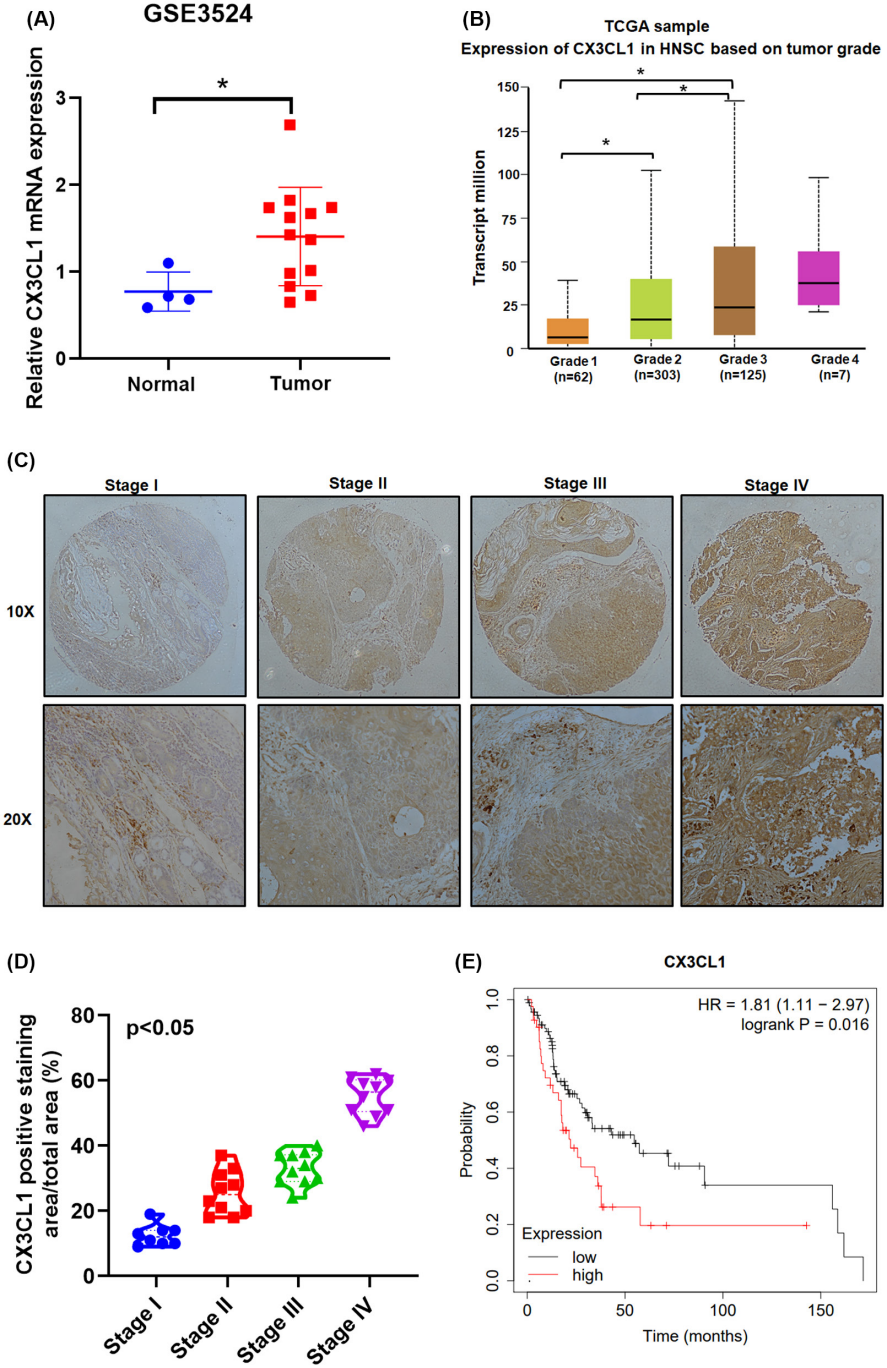
CX3CL1 was upregulated in oral squamous cell carcinoma (OSCC) tissue and associated with clinical disease stages in human OSCC. (A) Correlation CX3CL1 gene expression with the normal and tumour cells using GEO microarray 3524 OSCC tissue samples. (B) CX3CL1 expression profiles in 497 OSCC tissue specimens were analysed obtained from the Cancer Genome Atlas (TCGA) database. (C,D) OSCC specimens were subjected to IHC staining. (E) Kaplan–Meier survival analysis of the associations between high or low plasma levels of CX3CL1 expression and overall survival of OSCC patients. *p < 0.05 compared with controls.

To confirm the clinical significance of levels of CX3CL1 expression in OSCC, analysis of GEO microarray 3524 OSCC tissue samples revealed higher levels of CX3CL1 mRNA expression compared with levels in adjacent non‐tumour samples (*p* < 0.05; Figure 1A). As shown in Figure 1B, we found higher levels of CX3CL1 mRNA expression in TCGA sample of HNSC tissue compared with the oral mucosal tissue samples, and levels of CX3CL1 expression were significantly associated with clinical disease staging (*p* < 0.05). Results of IHC staining for levels of CX3CL1 in patients were with a higher‐grade OSCC than in those with a lower‐grade OSCC; the level of CX3CL1 expression was reflected by the tumour stage (Figure 1C,D). To assess the prognostic value of CX3CL1 expression in patients with OSCC, we investigated the associations among the CX3CL1 expression, overall survival (OS). We observed higher levels of CX3CL1 expression in patients with OSCC, which was also correlated with poor OS (*p* = 0.016) (Figure 1E). The results suggest that CX3CL1 is overexpressed in OSCC and is correlated with clinical stage and poor prognosis.

### 3.2 CX3CL1 upregulates OSCC cell migration and invasion

**FIGURE 2:**
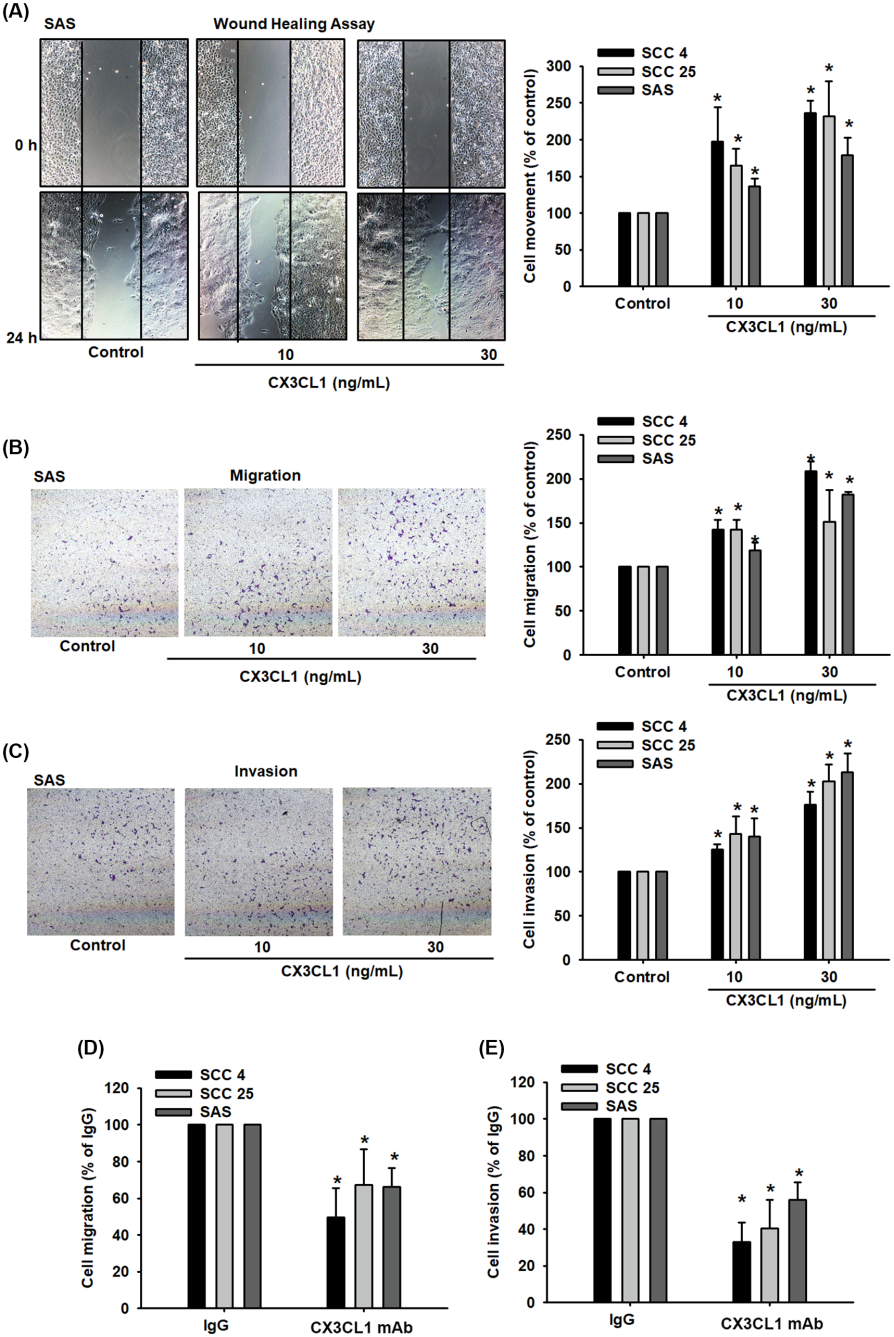
CX3CL1 upregulates human oral squamous cell carcinoma (OSCC) cell migration and invasion. (A‐C) OSCC cells were incubated with different concentrations of CX3CL1 for 24 h; then, cell migration was assessed using the (A) in vitro wound‐healing assay, (B,C) the Transwell assay. (D) Quantified result of cell migration with CX3CL1 neutralizing antibody. (E) Quantified result of cell invasion with CX3CL1 neutralizing antibody. Results are expressed as the mean ± SD of four independent experiments. *p < 0.05 as compared with controls.

CX3CL1 corresponds with node metastasis and early tumour stage in OSCC patients.^37^ Cancer metastasis involves sequential steps, such as cell movement, migration and invasion. In this study, we investigated the mechanism through which CX3CL1 regulates cell movement, cell migration, cell invasion and cell proliferation in OSCC. The wound‐healing assay and the Transwell migration assay revealed that CX3CL1 induced cell motility in the SCC4, SCC25 and SAS cell lines (Figure 2A,B). Furthermore, treatment with different concentrations of CX3CL1 enhanced cell invasiveness (Figure 2C). While simulation with CX3CL1 did not affect the proliferative capacity of oral cancer cells (Figure S1). However, these activities were inhibited when the cells were treated with CX3CL1 neutralizing antibody (Figure 2D,E). These findings confirmed that CX3CL1 promotes cell motility and metastatic activity of OSCC cells.

### 3.3 CX3CL1 upregulates cell migration via intercellular adhesion molecule‐1 (ICAM‐1) expression

**FIGURE 3:**
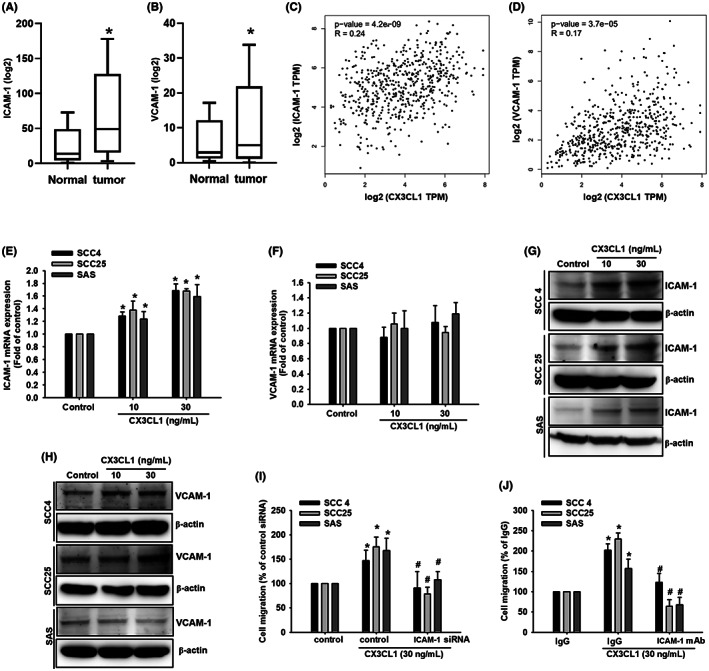
CX3CL1 activates tumour cell migration via the ICAM‐1 expression in human oral squamous cell carcinoma (OSCC) cells. (A) ICAM‐1 expression in the normal and tumour cells obtained from the TCGA dataset analysis. (B) VCAM‐1 expression in the normal and tumour cells obtained from the TCGA dataset analysis. (C) Correlation analysis of CX3CL1 and ICAM‐1 expression using the TIMER2.0 database. (D) Correlation analysis of CX3CL1 and VCAM‐1 expression using the TIMER2.0 database. (E) Quantified result of ICAM‐1 gene expression with CX3CL1 recombinant protein (30 ng/mL) treatment. (F) Quantified result of VCAM‐1 gene expression with CX3CL1 recombinant protein (30 ng/mL) treatment. (G) Protein expression of ICAM‐1 with concentration‐depended CX3CL1 treatment. (H) Protein expression of VCAM‐1 with concentration‐depended CX3CL1 treatment. (I) Quantified result of cell migration with ICAM‐1 siRNA and CX3CL1 recombinant protein (30 ng/mL) treatment. (J) Quantified result of cell migration with ICAM‐1 neutralizing antibody and CX3CL1 recombinant protein (30 ng/mL) treatment. Results are expressed as the mean ± SD of four independent experiments. *, *p* < 0.05 and #, *p* < 0.05 as compared to control and CX3CL1 treatment.

Cell adhesion molecules play a significant role in cell migration and cell‐matrix interactions, and tumour dissemination.^38^, ^39^ The expression of adhesion molecules enables the interaction between tumour cells and the surrounding stroma, resulting in tumour metastasis.^40^ Therefore, the function of ICAM‐1 and VCAM‐1 in OSCC cells was explored. Records from the TCGA dataset analysis revealed higher levels of ICAM‐1 and VCAM‐1 in tumour specimens compared with the normal specimens (Figure 3A,B). Furthermore, the correlations among ICAM‐1, VCAM‐1 and CX3CL1 were validated. TIMER2.0 analysis revealed a high positive correlation between gene expression of ICAM‐1 and CX3CL1 expression (*p* < 0.001, *r* = 0.24; Figure 3C), whereas a low positive correlation between gene expression of ICAM‐1 and CX3CL1 expression (*p* < 0.001, *r* = 0.17; Figure 3D). CX3CL1 treatment significantly increased ICAM‐1 but not VCAM‐1 mRNA and protein expression (Figure 3E,H). Furthermore, ICAM‐1 siRNA and ICAM‐1 neutralizing antibody treatment significantly reduced the ability of CX3CL1 to stimulate OSCC cell migration (Figure 3I,J). These findings indicated that CX3CL1‐mediated upregulation of OSCC cell migration was positively correlated with high ICAM‐1 expression.

### 3.4 CX3CL1 induces ICAM‐1 expression through CX3CR1 to promote tumour cell motility in OSCC

**FIGURE 4:**
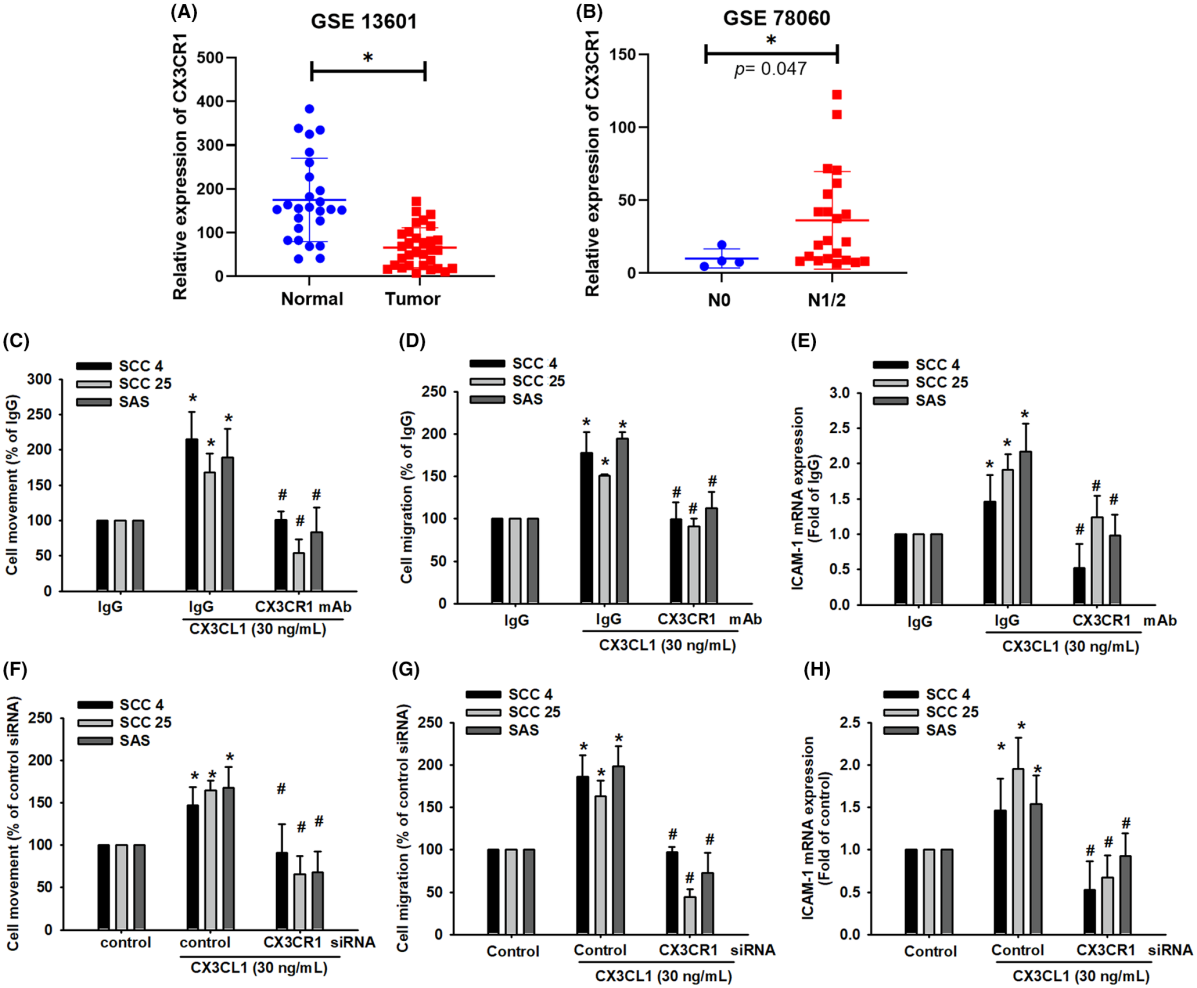
CX3CL1 upregulates cell motility and ICAM‐1 expression via its receptor CX3CR1. (A) Levels of CX3CR1 mRNA expression in normal tongue tissue and human oral squamous cell carcinoma (OSCC) tumour tissue were analysed using records from the GEO data set GSE13601. (B) Levels of CX3CR1 mRNA expression in different N stages of OSCC tumour tissue were analysed using records from the GEO data set GSE78060. (C) Quantified result of cell movement with CX3CR1 neutralizing antibody and CX3CL1 recombinant protein (30 ng/mL) treatment. (D) Quantified result of cell migration with CX3CR1 neutralizing antibody and CX3CL1 recombinant protein (30 ng/mL) treatment. (E) Quantified resulting of ICAM‐1 expression with CX3CR1 neutralizing antibody and CX3CL1 recombinant protein (30 ng/mL) treatment. (F) Quantified result of cell movement with CX3CR1 siRNA and CX3CL1 recombinant protein (30 ng/mL) treatment. (G) Quantified result of cell migration with CX3CR1 siRNA and CX3CL1 recombinant protein (30 ng/mL) treatment. (H) Quantified result of ICAM‐1 expression with CX3CR1 siRNA and CX3CL1 recombinant protein (30 ng/mL) treatment. Results are expressed as the mean ± SD of four independent experiments. *, *p* < 0.05 and #, *p* < 0.05 as compared to control and CX3CL1 treatment.

The CX3CR1 is a specific receptor for CX3CL1 and is involved in CX3CL1‐mediated cancer progress.^41^ To investigate associations between CX3CR1 expression in OSCC, we first analysed levels of CX3CR1 mRNA expression in normal and tumour tissue. Data from the GSE13601 microarray showed that, compared with the non‐tumour tissues, the mRNA expression of CX3CR1 was significantly downregulated in the tumour tissues (*p* < 0.01; Figure 4A). In addition, data from the GSE78060 microarray showed that CX3CR1 expression was positively correlated with N0 and N2 status (Figure 4B). To further investigate whether CX3CL1‐induced ICAM‐1 expression and cell migration are CX3CR1‐dependent mechanisms in OSCC, the CX3CR1 siRNA, and neutralizing antibody of CX3CR1 were used for evaluation. As shown in Figures 4C‐E, CX3CR1 neutralizing antibody could significantly suppress CX3CL1‐induced cell migration and mRNA expression of ICAM‐1. Similar results were observed in CX3CR1 siRNA (Figure 4F,G). These findings confirmed that CX3CL1‐induced cell migration and ICAM‐1 expression through binding to CX3CR1 in OSCC cells.

### 3.5 CX3CL1 induces cell motility by PLCβ, PKCα and c‐Src activation

**FIGURE 5:**
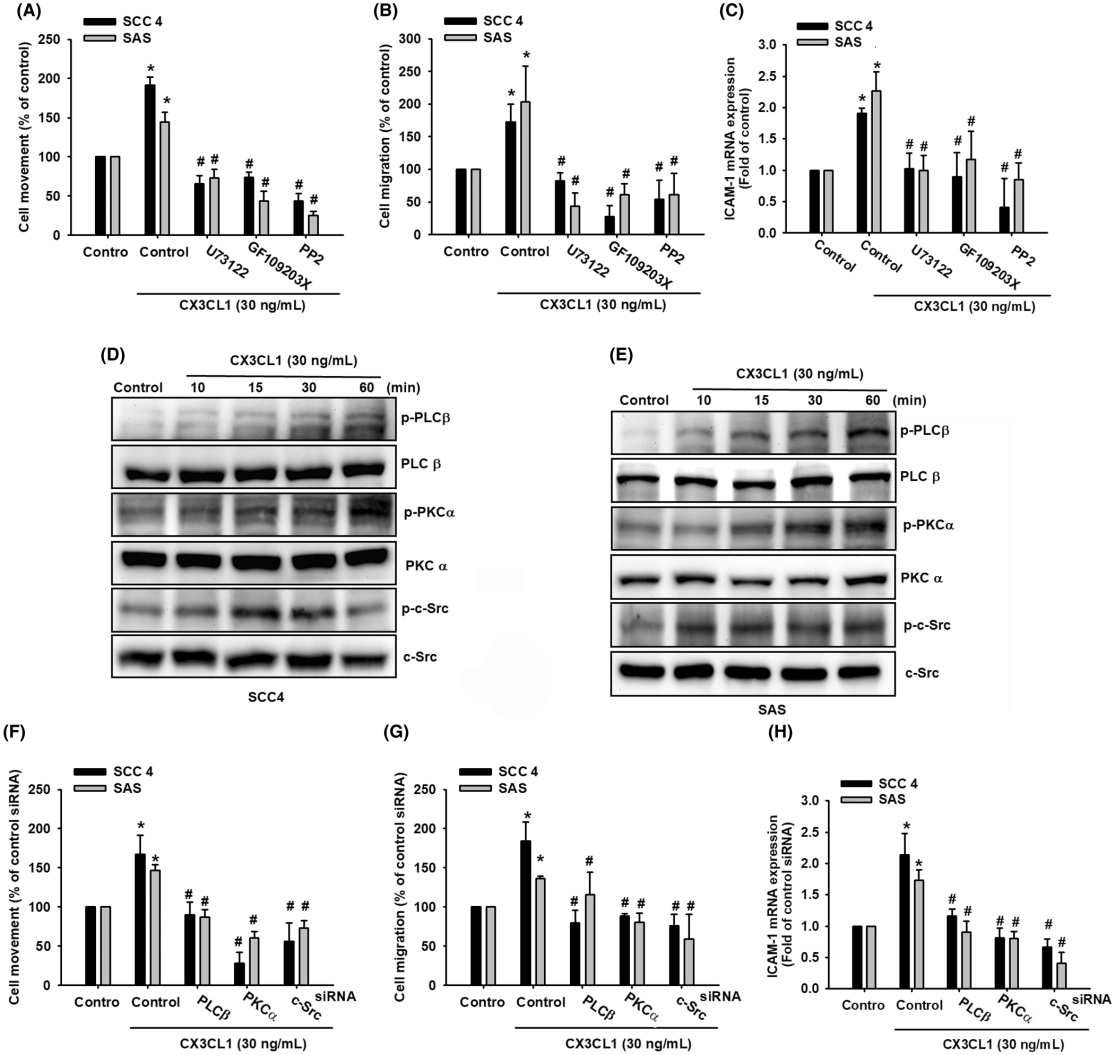
CX3CL1 triggers the phosphorylation of PLCβ/PKCα/c‐Src to signal the cell movement, migration and ICAM‐1 expression in human oral squamous cell carcinoma (OSCC) cells. (A‐C) Quantified result of cell movement, cell migration and ICAM‐1 expression with signalling inhibitors and CX3CL1 recombinant protein (30 ng/mL) treatment. (D‐E) Protein expression of phosphorylated PLCβ, PKCα and c‐Src with CX3CL1 recombinant protein (30 ng/mL) treatment in SCC4 cell and SAS cell. (F‐H) Quantified result of cell movement, cell migration and ICAM‐1 expression with PLCβ, PKCα or c‐Src siRNA and CX3CL1 recombinant protein (30 ng/mL) treatment. Results are expressed as the mean ± SD of four independent experiments. *, *p* < 0.05 and #, *p* < 0.05 as compared to control and CX3CL1 treatment.

Studies indicated that the activation of PLCβ, PKCα or c‐Src has a critical role in tumour growth, cell migration, invasiveness and metastasis in breast cancer, colon carcinoma, lung squamous cell carcinoma and hepatocellular carcinoma cells.^42^, ^43^, ^44^, ^45^ Here, we investigated whether CX3CL1 activated these signalling pathways in OSCC. The results indicated that pre‐treatment with PLCβ, PKCα and c‐Src inhibitors (U73122, GF109203X and pp2) reversed CX3CL1‐promoted cell migration and ICAM‐1 expression in OSCC (Figure 5A‐C). Moreover, incubation with CX3CL1 obviously induced phosphorylation of PLCβ, PKCα and c‐Src (Figure 5D,E). Finally, we certified our finding by using siRNA to suppress these pathways activation, and the data showed that transfection with PLCβ, PKCα and c‐Src siRNA apparently reversed cell migration and ICAM‐1 expression after CX3CL1 incubation (Figure 5E‐G). Similarly, the CX3CL1‐induced phosphorylation of PLCβ, PKCα and c‐Src was reversed by pre‐treatment with CX3CR1 neutralized antibody (Figure S2). These results suggest that the PLCβ/PKCα/c‐Src signalling pathways contribute to CX3CL1‐promoted cell migration in OSCC.

### 3.6 AP‐1 activation and c‐Jun phosphorylation are involved in the CX3CL1‐induced tumour cell motility and ICAM‐1 expression in OSCC cells

**FIGURE 6:**
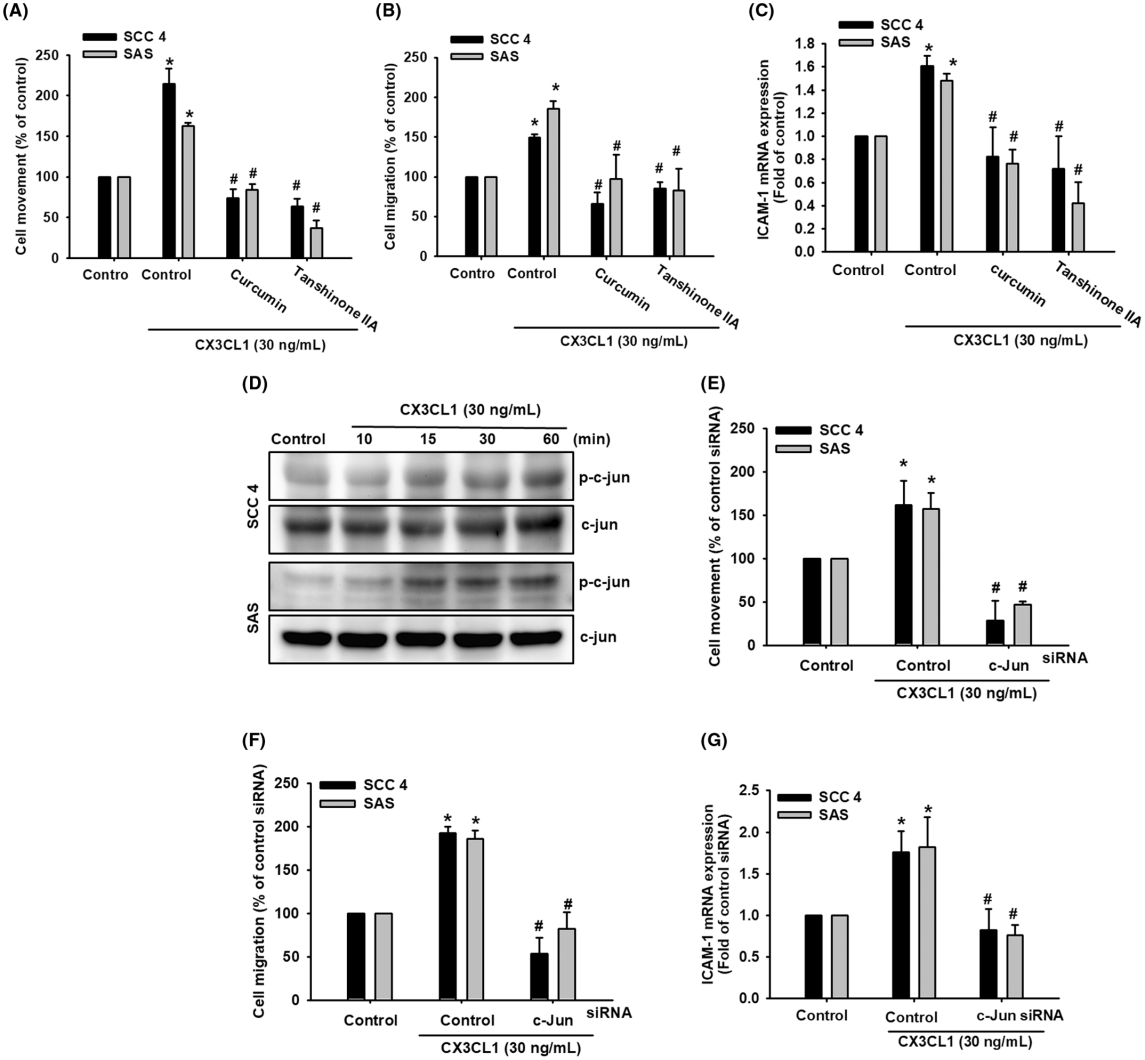
CX3CL1 promotes the phosphorylation of c‐Jun to upregulate the cell motility and ICAM‐1 expression in human oral squamous cell carcinoma (OSCC) cells. (A) Quantified result of CX3CL1‐induced cell movement with c‐Jun inhibitors (B) Quantified result of CX3CL1‐induced cell migration with c‐Jun inhibitors (C) Quantified result of CX3CL1‐induced ICAM‐1 expression with c‐Jun inhibitors (D) Protein expression of phosphorylated c‐Jun with CX3CL1 recombinant protein (30 ng/mL) treatment. (E) Quantified result of CX3CL1‐induced cell movement with c‐Jun siRNA treatment. (F) Quantified result of CX3CL1‐induced cell migration with c‐Jun siRNA treatment. (G) Quantified result of CX3CL1‐induced ICAM‐1 expression with c‐Jun siRNA treatment. Results are expressed as the mean ± SD of four independent experiments. *, *p* < 0.05 and #, *p* < 0.05 as compared to control and CX3CL1 treatment.

The nuclear transcription factor AP‐1, associated with cell proliferation and tumorigenesis, has been identified as a transcription regulator of ICAM‐1 expression.^46^ To examine the role of the AP‐1 binding site in CX3CL1‐induced cell motility and ICAM‐1 expression in OSCC, pre‐treating cells with AP‐1 inhibitors (curcumin and tanshinone IIA) reduced CX3CL1‐induced cell movement, migration and ICAM‐1 expression (Figure 6A‐C). Moreover, our results also showed that levels of phosphorylated c‐Jun were upregulated after 30–60 min of CX3CL1 treatment (Figure 6D). To further demonstrate that c‐Jun can mediate CX3CL1‐induced cell migration and ICAM‐1 expression, siRNA transfection was performed to silence c‐Jun mRNA in OSCC cells. The results demonstrated that c‐Jun siRNA transfection suppressed CX3CL1‐induced cell migration and ICAM‐1 mRNA expression (Figure 6E‐G). These findings unveiled that the downregulated CX3CL1‐induced cell motility and ICAM‐1 expression were positively correlated with the AP‐1 activation in OSCC.

**FIGURE 7:**
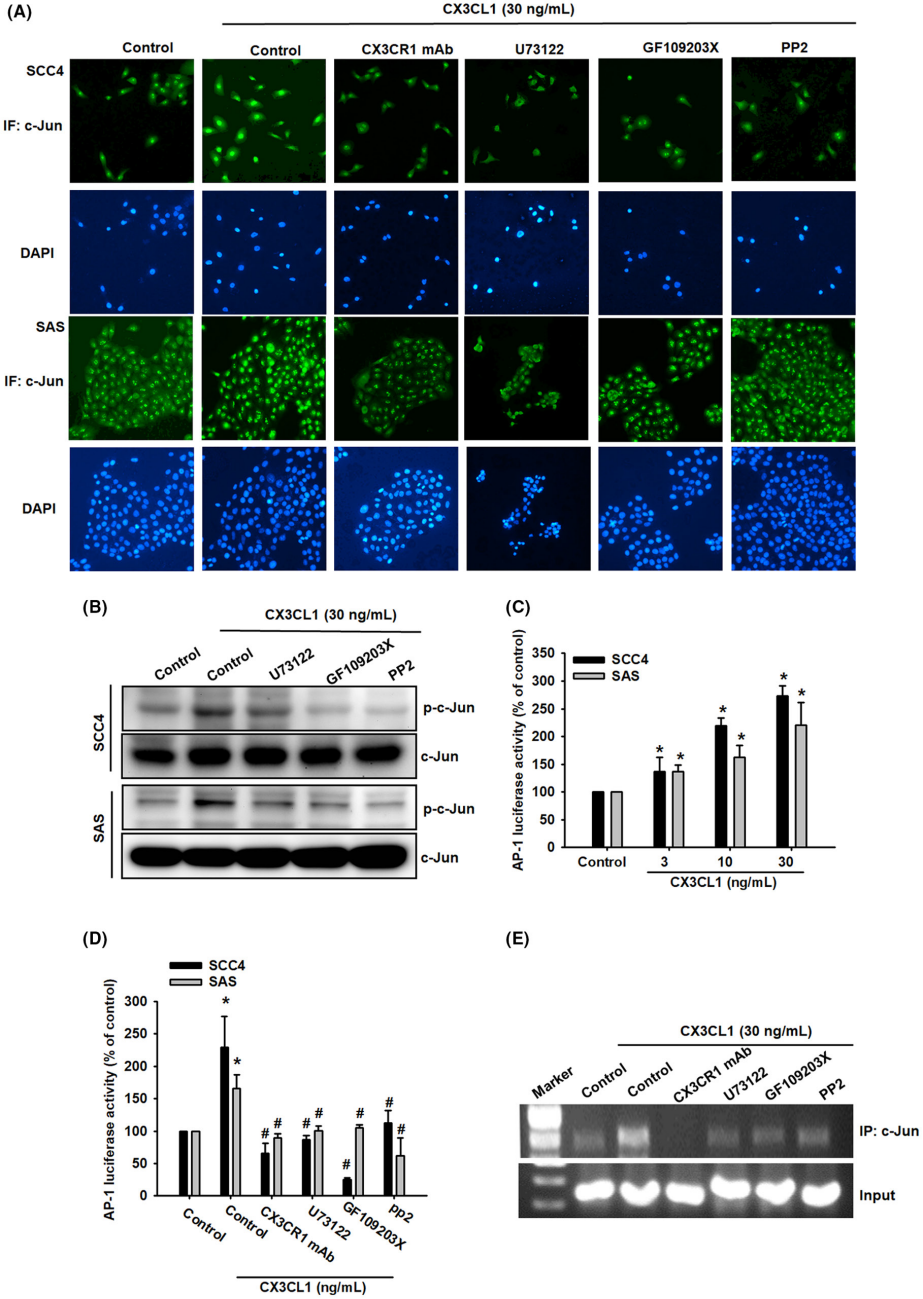
CX3CL1 promotes the translocation of c‐Jun and promoter binding of AP‐1 formation in human oral squamous cell carcinoma (OSCC) cells. (A) Immunofluorescent staining showed that c‐Jun translocated into the nucleus after cell were treated with CX3CL1; nuclear translocation was prevented when the cells were pre‐treated with CX3CR1 mAb, U73122, GF109203 or PP2. (B) Quantified analysis of c‐Jun activation with CX3CL1 using Western blot. (C) CX3CL1 treatment upregulated AP‐1 luciferase activity. (D) Quantified analysis of AP‐1 luciferase activity with specific inhibitors and CX3CL1 recombinant protein (30 ng/mL) treatment. (E) Chromatin immunoprecipitation analysis of c‐Jun and AP‐1 promoter binding site with neutralizing antibody and specific inhibitors. Results are expressed as the mean ± SD of four independent experiments. *, *p* < 0.05 and #, *p* < 0.05 as compared to control and CX3CL1 treatment.

We also checked whether CX3CR1, PLCβ, PKCα and c‐Src were the upstream regulator of AP‐1, with pre‐treatment of CX3CR1 neutralized antibody, PLCβ, PKCα or c‐Src inhibitors, the CX3CL1‐induced c‐Jun nuclear translocation, representing for AP‐1 activation, was reversed by pre‐treatment with CX3CR1 neutralized antibody, PLCβ, PKCα and c‐Src inhibitors (Figure 7A), as well as c‐Jun phosphorylation (Figure 7B). Finally, to further examine the transcriptional activation of AP‐1 is responsible for CX3CL1 effects, the luciferase reporter assay was performed. The AP‐1 activity was significantly increased after CX3CL1 treatment (Figure 7C). However, in the presence with pathway inhibitors of CX3CR1, PLCβ, PKCα and c‐Src, the AP‐1 activity was reversed after CX3CL1 treatment (Figure 7D). In addition, transcriptional activation of AP‐1 was further investigated whether it participates in CX3CL1‐promoted ICAM‐1 expression. The chromatin immunoprecipitation (ChIP) assay was examined for evaluation and it was suggested that these pathway inhibitors could abolish the binding ability of CX3CL1‐induced c‐Jun to the AP‐1 binding element on the ICAM‐1 promoter (Figure 7E). These results demonstrated that the CX3CL1‐driven CX3CR1, PLCβ, PKCα and c‐Src pathway is involved in the regulation of AP‐1 expression and nuclear translocation, and subsequently affected the expression of AP‐1‐dependent ICAM‐1.

## 4 DISCUSSION

Human oral squamous cell carcinoma is one of the common malignant tumours occurring in the oral cavity with a low 5‐year survival rate, especially in the advanced stage. Its low survival rate is associated with regional metastasis involving penetration of cervical lymph nodes and distant metastasis resulting from the invasion‐metastasis cascade involving activation of various kinases and molecules.^47^, ^48^ Understanding the pathogenesis of cancer metastasis at the molecular level and kinase pathway could offer approaches for therapeutic targeting.^49^, ^50^ The present study demonstrated that the CX3CL1‐CX3CR1 axis mediates ICAM‐1 expression and subsequently induces tumour migration and invasion in OSCC cells, suggesting that CX3CL1 is a crucial indicator in determining the prognosis of tumour metastasis and provides a novel tumour therapeutic target in human oral squamous cell carcinoma.

CX3CL1‐CX3CR1 axis facilitates cell–cell interaction and communication or exerts paracrine and endocrine effects on different tissues, including tumour invasiveness and metastasis.^12^, ^13^, ^14^ CX3CL1 plays a vital role in regulating cell adhesion, migration and survival of human cancer cells. CX3CL1‐CX3CR1 also played an essential role in malignant tumours by regulating cellular functions associated with metastasis. The highly invasive OSCC cells, such as SAS‐Hl cell line, expressed upregulation of the chemokine receptor CX3CR1, especially in the transforming growth factor (TGF)‐β1‐regulated microenvironment and then potentiated CX3CL1‐induced directional migration and adhesion. These data implied that CX3CR1 was associated with N stages and cancer metastasis.^51^ The clinical significance of levels of CX3CL1 expression in OSCC and low survival rate was confirmed from the online database. Furthermore, the present study demonstrates that CX3CL1 promotes OSCC cell migration and invasion through ICAM‐1 expression, indicating that CX3CL1 expression is an appropriate therapeutic target in OSCC.

VCAM‐1 and ICAM‐1 play critical roles in cell adhesion to the endothelium and tumour development, cell proliferation, invasion and angiogenesis.^52^ ICAM‐1 promotes tumour relapse and metastasis in colorectal cancer, lymphoma, hepatocellular carcinoma cells and epithelial tumorigenesis.^53^, ^54^, ^55^ VCAM‐1 has also been reported to mediate lung and bone metastasis.^56^ Therefore, the correlation between CX3CL1 and ICAM‐1 or VCAM‐1 is explored in the present study, showing that only CX3CL1‐induced ICAM‐1 expression is observed, indicating that ICAM‐1 plays a vital role in CX3CL1‐induced cancer metastasis. ICAM‐1 mAb and siRNA result in a downregulation of CX3CL1‐induced tumour metastasis in OSCC cells. This study proves that the CX3CL1‐CX3CR1 axis is vital in promoting ICAM‐1‐regulated tumour metastasis. Consequently, targeting the CX3CL1‐CX3CR1 axis and ICAM‐1 expression is the potential therapeutic goal for ameliorating oral‐related tumours.

The ICAM‐1 expression is modulated via the gene transcription on ICAM‐1 promoter, involving several transcription factors and various signalling pathways.^57^, ^58^, ^59^ Upregulated ICAM‐1 expression required the activation of PLCβ via tyrosine kinase to induce PKCα and c‐Src, allowing the transcriptional factors to bind on the AP‐1 promoter binding sites for tumour cell motility.^58^, ^59^ The present study demonstrates that the CX3CL1‐CX3CR1 axis promoted the phosphorylation of PLCβ, PKCα and c‐Src in OSCC cells. Moreover, the PLCβ/PKCα/c‐Src inhibitors and transfection of siRNAs downregulate the CX3CL1‐induced ICAM‐1 expression and cell migration. Thus, the present study demonstrates PLCβ/PKCα/c‐Src pathway is essential for CX3CL1‐induced cell migration and ICAM‐1 expression in OSCC cells.

The Fos and Jun family members bind to the AP‐1 recognizing sites on the ICAM‐1 promoter in homodimer or heterodimer forms, resulting in the transcription of AP‐1 responsive genes, which are highly expressed and active in melanoma cells.^60^ Similar results were observed in the present study. CX3CL1 promoted c‐Jun phosphorylation and c‐Jun translocation into the nucleus through CX3CR1/PLCβ/PKCα/c‐Src pathway in OSCC cells. Cells pre‐treating c‐Jun siRNA abolishes CX3CL1‐induced cell migration and ICAM‐1 expression, demonstrating that c‐Jun phosphorylation mediates CX3CL1‐induced cancer metastasis and ICAM‐1 expression. In addition, CX3CL1 promotes the binding of c‐Jun to the AP‐1 recognizing sites on the ICAM‐1 promoter. The binding formation of c‐Jun to AP‐1 recognizing sites was debilitated by CX3CR1 mAb and PLCβ/PKCα/c‐Src inhibitors. The present study demonstrates that CX3CL1 acts through the CX3CR1/PLCβ/PKCα/c‐Src/c‐Jun/AP‐1 pathway to activate ICAM‐1 expression and metastasis in OSCC cells. Taken together, we suggested a novel treatment and a prognostic indicator targeting CX3CL1‐induced tumour motility of human oral squamous cell carcinoma. CX3CL1, as a prognosis indicator, promoted tumour cell migration, invasion and ICAM‐1 expression by activating CX3CR1/PLCβ/PKCα/c‐Src/c‐Jun/AP‐1 pathway.

## 5 CONCLUSIONS

In the present study, high levels of CX3CL1 expression were evidenced in OSCC cells. A molecular mechanism of CX3CL1‐induced OSCC cell migrations and cell invasion was presented through the upregulation of ICAM‐1 expression. CX3CL1 upregulates ICAM‐1 expression through the proposed CX3CR1/PLCβ/PKCα/s‐Src/AP‐1 signalling pathway and subsequently promotes tumour metastasis.

## AUTHOR CONTRIBUTIONS

**Chia‐Yu Wu:** Methodology (equal); visualization (equal); writing – original draft (equal). **Pei‐Wen Peng:** Writing – original draft (equal); writing – review and editing (equal). **Ting‐Yi Renn:** Data curation (equal); methodology (equal). **Chia‐Jung Lee:** Data curation (equal); methodology (equal). **Tsung‐Ming Chang:** Conceptualization (equal); data curation (equal); formal analysis (equal); investigation (equal); methodology (equal); supervision (equal). **Augusta I‐Chin Wei:** Data curation (equal); methodology (equal). **Ju‐Fang Liu:** Conceptualization (equal); resources (equal); supervision (equal); writing – original draft (equal); writing – review and editing (equal).

## CONFLICT OF INTEREST STATEMENT

The authors declare that they have no conflict of interest.

## Supporting information


Figure S1

Figure S2

## Data Availability

The datasets generated for this study can be accessed upon request to the corresponding author.
