# Supplementary figures and images for: CX3CL1 induces cell migration and invasion through ICAM‐1 expression in oral squamous cell carcinoma cells
Source: J Cell Mol Med. 2023 Apr 21;27(11):1509–22. doi: 10.1111/jcmm.17750 (PMC10243164; doi:10.1111/jcmm.17750)

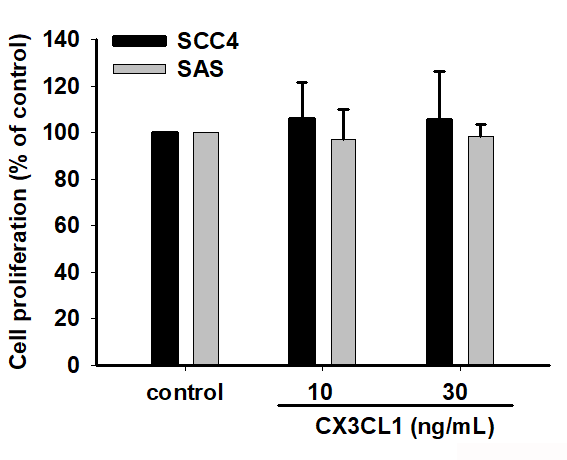

Supplement: Supplementary file 1 — Figure S1 [file JCMM-27-1509-s001.tif]

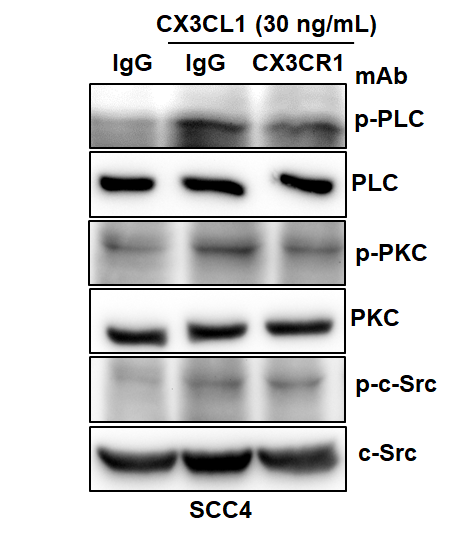

Supplement: Supplementary file 2 — Figure S2 [file JCMM-27-1509-s002.tif]
